# Critical Assessment of Protein Intrinsic Disorder Round 3 ‐ Predicting Disorder in the Era of Protein Language Models

**DOI:** 10.1002/prot.70045

**Published:** 2025-08-26

**Authors:** Mahta Mehdiabadi, Alessio Del Conte, Maria Victoria Nugnes, Maria Cristina Aspromonte, Silvio C. E. Tosatto, Damiano Piovesan

**Affiliations:** ^1^ Department of Biomedical Sciences University of Padova Padua Italy; ^2^ Institute of Biomembranes, Bioenergetics and Molecular Biotechnologies, National Research Council (CNR‐IBIOM) Bari Italy

**Keywords:** CAID, critical assessment, DisProt, intrinsic disorder prediction, intrinsically disordered proteins

## Abstract

Intrinsic disorder (ID) in proteins is a complex phenomenon, encompassing a continuum from entirely disordered regions to structured domains with flexible segments. The absence of a ground truth for all forms of disorder, combined with the possibility of structural transitions between ordered and disordered states under specific conditions, makes accurate prediction of ID especially challenging. The Critical Assessment of Protein Intrinsic Disorder (CAID) evaluates ID prediction methods using diverse benchmarks derived from DisProt, a manually curated database of experimentally validated annotations. This paper presents findings from the third round (CAID3), in which 24 new methods were assessed along with the predictors from previous rounds. Compared to CAID2, the top‐performing methods in CAID3 demonstrated significant gains in average precision: over 31% improvement in predicting linker regions, and 15% in disorder prediction. This round introduces a new binding sub‐challenge focused on identifying binding regions within known IDR boundaries. The results indicate that this task remains challenging, highlighting the potential for improvement. The top‐performing methods in CAID3 are mostly new and commonly used embeddings from protein language models (pLMs), underscoring the growing impact of pLMs in tackling the complexities of disordered proteins and advancing ID prediction.

## 1 Introduction

Intrinsically disordered proteins and regions (IDPs/IDRs) are characterized by amino acid sequences that lack a stable tertiary structure under physiological conditions [1]. Instead, they are defined by a heterogeneous ensemble of conformations [2].

Experimental techniques to study IDPs typically capture only the average behavior of a dynamic ensemble [3]. For example, NMR spectroscopy offers valuable conformational constraints, but it does not provide detailed insight into the temporal aspects of protein dynamics [4]. Moreover, many IDPs exhibit context‐dependent behavior, transitioning between ordered and disordered states in response to factors such as binding partners, pH changes, and other environmental conditions [5].

The Critical Assessment of Protein Intrinsic Disorder (CAID) was established in 2018 as a community‐based effort to address the challenges of predicting intrinsic disorder [6, 7]. The biennial challenge focuses on analyzing the problem of identifying IDR positions within the protein sequence by assessing the prediction methods in terms of performance and usability. In addition to disorder prediction, CAID features sub‐challenges focused on binding‐site and linker prediction in IDRs. The reference set provided by the DisProt database [8] contains several benchmarks developed to test the accuracy and performance of the methods in different scenarios.

To support the long‐term goals of the CAID community challenge, the CAID prediction portal [9] was created as a platform for running and executing CAID predictors on any given sequence. The portal provides users with disorder prediction results from various methods and helps them determine which predictor is best suited for their specific task within a reasonable timeframe.

Disorder predictors utilize diverse approaches to predict IDRs. They range from energy‐based methods [10, 11] to machine learning techniques [12, 13]. Recently, protein Language Models (pLMs) [14, 15, 16] have emerged as a powerful tool to tackle protein‐related tasks. Due to their large‐scale pretraining, these models effectively capture the residue patterns shaped by evolutionary processes [16, 17] and learn implicit functional and structural information from sequence data [18, 19, 20]. As illustrated in this work, many of the latest disorder predictors use pLMs in IDR prediction, indicating that pLMs could capture the intrinsic disorder encoded in the sequence.

This work presents the results of the third round of CAID. We evaluate 24 new methods participating in CAID3, alongside predictors from previous rounds, totaling 68 methods, on a set of proteins for which disorder annotations were unavailable when methods were developed. We compare the performance of predictors, highlighting that the average precision (APS) of the top methods in the linker and disorder categories has improved by more than 31% and 15%, respectively. Furthermore, we present a new variation of the binding prediction challenge, where benchmark sequences are confined to IDRs. Notably, the top‐ranked methods in most of the benchmarks are newly introduced in CAID3, with many employing protein language models (pLMs) to predict intrinsic disorder from sequence.

## 2 Materials and Methods

### References

2.1

**FIGURE 1:**
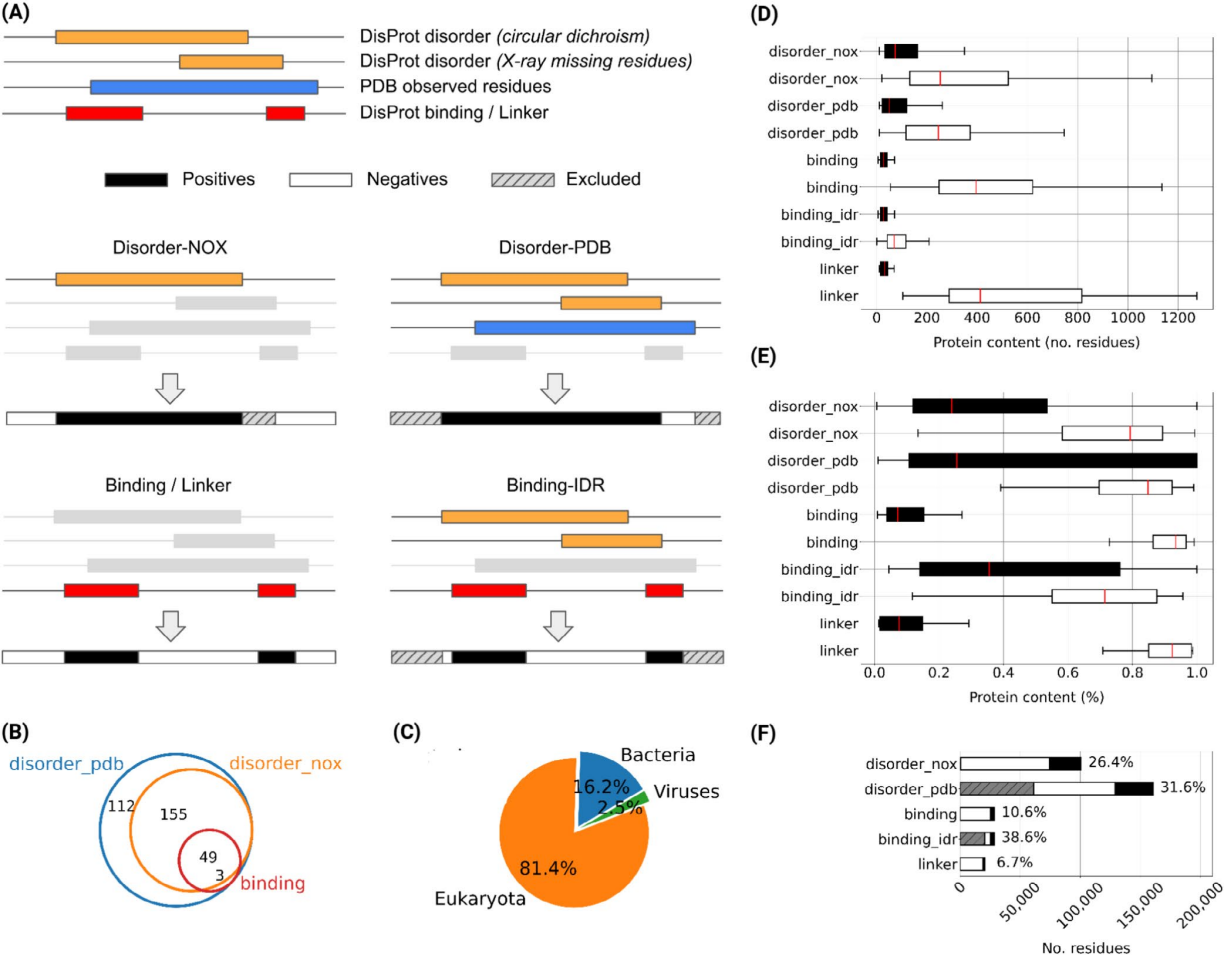
CAID3 dataset statistics. (A) Definitions of references. (B) The Venn diagram of proteins in the benchmarking references. (C) Distribution of proteins across the primary domains of life for the Disorder‐PDB dataset. (D) The distribution of positive and negative classes at the protein level by the number of residues, and (E) as a percentage normalized by protein length. (F) Residue classifications: The fraction of positive residues, considering non‐excluded residues, is reported on the right side of the bars. Excluded residues are 541 (0.5%) for Disorder‐NOX, 61,183 (38.1%) for Disorder‐PDB and 20,522 for Binding‐IDR (72.6%).

Reference proteins are obtained by calculating the delta between the public and private versions of the DisProt database between December 2023 and June 2025, respectively. DisProt is a manually curated database in which annotations are obtained from the literature. In most cases, they are validated by more than one experimental evidence, providing a reliable source of disorder ground truth. In DisProt, IDRs are defined as sequence segments of at least 10 residues likely to be associated with a biological function, excluding short loops connecting secondary structure elements. Figure 1 shows the generation of the reference sets and their statistics.

The primary disorder benchmarking reference is called “Disorder‐NOX”. In this dataset, all disorder‐annotated residues in DisProt excluding X‐ray missing residues are considered positive, and the rest is negative (Figure 1A). The missing residues are excluded from the evaluation using the structural information from the PDB [21]. Excluding missing residues decreases the number of protein targets, but the average number of positive residues per protein increases. This is due to the larger size of disorder regions detected by experimental techniques other than X‐ray. Indeed, X‐ray cannot be used to infer disorder from largely or fully disordered proteins, by definition.

Since DisProt annotations originate from time‐consuming and technically challenging experiments, the annotations for the entire disorder or binding regions in the sequence may not be available. The “Disorder‐PDB” reference addresses this incompleteness of annotations. In this dataset, all disorder‐annotated residues in DisProt are considered as positive, but the negative residues are constrained to the observed regions (resolved coordinates) in PDB. This dataset is conservative and considered more reliable as it excludes uncertain residues without structural or disorder annotation.

As in CAID2 [7], the “Binding” reference includes proteins with at least one binding region annotated in DisProt. Binding residues are labeled as positive, while all other residues (disordered or structured) are considered negative. To address the label imbalance (Figure 1D,E), we introduce an additional benchmark, “Binding‐IDR,” which uses the same proteins and positive content but limits negatives to disordered residues not annotated as binding. This benchmark evaluates whether methods can identify binding regions from non‐binding IDRs, whereas the original benchmark assesses the predictions across the entire sequence. The definitions of binding include all types of binding, for example, nucleic acid, protein binding, etc.

The “Linker” reference is constructed as in CAID2, and includes proteins with at least one linker region as annotated by DisProt (Figure 1A). The linker regions are defined in DisProt as unstructured regions, providing separation and permitting movement between adjacent functional regions, such as structured domains.

Disorder‐NOX and Disorder‐PDB contain 204 and 319 sequences, respectively; Binding and Binding‐IDR each include 52 sequences, while Linker has 31. The Venn diagram of references is presented in Figure 1B.

The targets in CAID3 originate from diverse organisms (Figure 1C). Within the Disorder‐PDB superset, 232 sequences (72.7%) are from eukaryotes, 80 (25.0%) from bacteria, 7 (2.2%) from viruses, and none from archaea. The target proteins differ from the “old” DisProt (December 2023), having a mean and median local sequence identity of 31.6% and 23.9%, respectively.

The total number of positive residues is 26,367 (26.4%), 31,401 (31.6%), 2,991 (10.6%), 2,991 (38.6%), and 1,379 (6.7%) for the Disorder‐NOX, Disorder‐PDB, Binding, Binding‐IDR, and Linkers references, respectively (Figure 1F).

**FIGURE 2:**
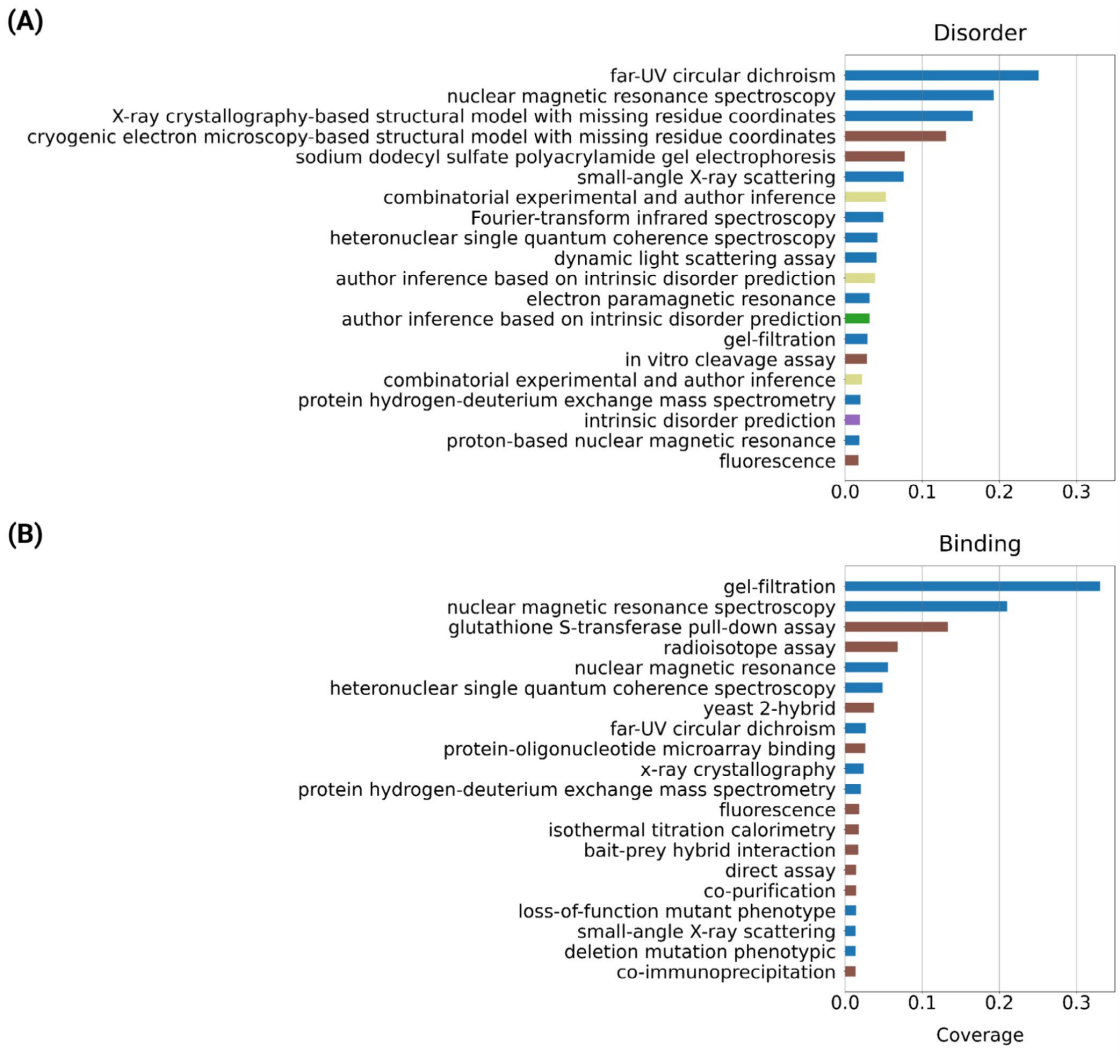
The fraction of disordered residues in the Disorder‐PDB reference (A), and Binding reference (B), covered by specific experimental evidence. Some regions may be identified by multiple experiments. Only the top 20 experimental methods are reported. Method names are shortened; For instance, “intrinsic disorder prediction” corresponds to “intrinsic disorder prediction evidence used in the manual assertion”. Colors reflect the grouping of methods based on the Evidence and Conclusion Ontology (ECO) hierarchy.

Figure 2A, illustrates the fraction of residues covered by experimental techniques, defined by Evidence and Conclusion Ontology (ECO) [22] terms. In the Disorder‐PDB reference, “*far‐UV circular dichroism*” accounts for over 25% of the disordered residues, followed by “*nuclear magnetic resonance spectroscopy*” and “*X‐ray crystallography*”. Figure 2B displays the same statistics for binding reference. In contrast to disorder annotations, binding annotations include only experimental and direct assay evidence, and no “manual assertions”.

### 2.2 Predictions and Containers

Predictors' software were containerized using Singularity (https://sylabs.io/) to standardize the input/output, ensure reproducibility, and eliminate manual installation. This approach bundled the software and dependencies for consistent deployment and updates. Scripts standardized input/output before and after execution, creating an interface for the predictor. Each predictor processed a FASTA file with multiple sequences, generating single or multiple outputs per sequence while recording execution times.

For predictors requiring additional inputs such as PSI‐BLAST, HHblits, or SPIDER2 results, these were precomputed for all sequences, with their processing time included in the total runtime. Similarly, for predictors relying on pLM embeddings, we generated these representations with a custom script and added their generation time to the method's runtime. Each predictor was allocated a maximum of 4 h per sequence, 24 CPU cores, and 47 GB of RAM.

### 2.3 Evaluation

CAID3 adopts the same evaluation approach as CAID2. Briefly, given a protein sequence, the predictors are required to assign a score and a state to each residue for its propensity to be intrinsically disordered, binding‐site, and linker for the disorder, binding, and linker prediction challenges, respectively. Scores are decimal values, while states are binary labels indicating whether a residue is structured or disordered. If scores are unavailable, states are used as proxies; when states are absent, they are derived by applying a threshold to the scores. Scores are rounded to three decimal places to define 1000 potential thresholds.

The primary evaluation metrics are *F*_max_, average precision score (APS), and AUC, where *F*_max_ represents the maximum point on the precision‐recall curve, and AUC measures the area under the receiver operating characteristic (ROC) curve. APS was calculated as the mean precision along the precision‐recall curve. It reflects the area under the precision‐recall curve, providing a more robust measure of a method's ability to prioritize disordered regions than *F*_max_.

In CAID3, we used the DeLong test [23] to compare the AUC of predictors and assess their performance differences. The DeLong test is a non‐parametric statistical test that compares the AUC (Area Under the Curve) scores of two classifiers. It estimates the variance of AUC using covariance calculations, providing a more efficient and accurate alternative to bootstrapping. The test computes a *Z*‐score based on the difference in AUCs and their variances, then derives a *p*‐value to determine if the difference is statistically significant. This helps us assess whether one model meaningfully outperforms another or if the observed difference is due to random variation.

### 2.4 AlphaFold Disorder and Binding Prediction

In CAID2, the AlphaFold‐disorder package [24] was developed as a baseline to predict intrinsic disorder, utilizing structures publicly available in AlphaFoldDB [25]. Briefly, it generates three types of predictions: (i) AlphaFold‐pLDDT, which is the 1‐pLDDT score; (ii) AlphaFold‐rsa, which predicts the relative solvent accessibility (RSA) over a local window centered on the residue to be predicted; and (iii) AlphaFold‐binding, which identifies regions with high RSA and high pLDDT.

AlphaFold3 [26] was released in May 2024. Following the success of AlphaFold2 [27] in disorder prediction [7, 24], we developed the AlphaFold3‐disorder package, which produced three types of prediction: AlphaFold3‐rsa, AlphaFold3‐pLDDT, and AlphaFold3‐binding, using the same approaches. Protein structures for all reference sets were downloaded from the web server. This method is not included in the execution runtime assessment.

To maintain consistency with CAID2, we kept the naming convention for AlphaFold2 predictions unchanged (i.e., AlphaFold‐rsa, AlphaFold‐pLDDT, and AlphaFold‐binding). For 25 proteins not available in AlphaFoldDB, structures were generated using ColabFold with Amber relaxation [28, 29].

## 3 Results and Discussion

In CAID3, as in previous rounds, participants were required to submit their prediction software to the assessors, containerized using either Singularity or Docker. The assessors ran the predictors and generated the results for a set of proteins for which disorder annotations were not previously available.

Twenty four new predictors were submitted to CAID3 (two methods collected from the literature, which were trained before the challenge's timeline). Most of these programs generated multiple outputs, resulting in 46 different predictor “flavors” corresponding to the different variations of the predictor. All the methods participating in CAID1 and CAID2 are included in the evaluations. Overall, in CAID3, we executed and assessed 68 software programs that generated 117 flavors.

In this edition, 17 out of 24 (70%) new methods were submitted as containers. This marks a significant shift from CAID1 and CAID2, where none of the methods were submitted containerized. This progress highlights the community's growing commitment to standardization and reproducibility, one of the key objectives of CAID since its establishment in 2018.

Here, we present the results of the CAID3 challenge. We will point out the improvements in CAID3 and discuss the role of protein language models in IDR prediction. Complete results, including interactive figures, precision‐recall and ROC curves, and timings of all the methods across all benchmarks, can be found on the CAID challenge results page (https://caid.idpcentral.org/challenge/results).

### 3.1 Disorder Prediction

The top rankings for Disorder‐NOX and Disorder‐PDB are presented in Tables 1 and 2, sorted by their AUC. While the top method remains unchanged, rankings sorted by APS differ significantly (see CAID challenge results).

**TABLE 1:** Top 10 ranking of disorder‐NOX challenge in CAID 3.

Disorder‐NOX					
Predictor	New	pLM	AUC	APS	Cov
ESMDisPred‐2PDB	×	×	0.885	0.754	0.887
ESMDisPred‐1	×	×	0.876	0.745	0.882
ESMDisPred‐2	×	×	0.872	0.743	0.887
DisoFLAG‐IDR [30]	×	×	0.868	0.714	0.975
DisorderUnetLM [31]	×	×	0.862	0.700	0.995
flDPnn3a	×	×	0.860	0.700	1.000
UdonPred‐combined	×	×	0.854	0.654	0.985
DisPredict3 [32]		×	0.854	0.633	0.950
rawMSA [33]			0.850	0.671	1.000
rawMSA‐disorder	×		0.847	0.640	0.990

*Note*: New indicates whether it was submitted to CAID 3. pLM denotes the use of protein language model information. AUC is the area under the receiver operating characteristic (ROC) curve, and APS represents the average precision score along the precision‐recall curve. Cov refers to the proportion of the reference proteins the method successfully covers. Methods are sorted based on the AUC.

**TABLE 2:** Top 10 ranking of disorder‐PDB challenge.

Disorder‐PDB					
Predictor	New	pLM	AUC	APS	Cov
PUNCH2 [34]	×	×	0.955	0.928	1.000
PUNCH2‐Light [34]	×	×	0.953	0.925	1.000
AlphaFold‐rsa [24]			0.950	0.921	1.000
SPOT‐Disorder2 [35]			0.949	0.920	0.934
AlphaFold3‐rsa	×		0.947	0.912	1.000
LMDisorder [36]	×	×	0.937	0.621	1.000
ESMDisPred‐2PDB	×	×	0.937	0.893	0.890
PredIDR2‐Seq‐Art [37]	×		0.939	0.809	1.000
PredIDR2‐Prof‐Art [37]	×		0.936	0.884	1.000
PredIDR2‐Prof‐Rnd [37]	×		0.934	0.829	1.000

*Note*: New indicates whether it was submitted to CAID3. pLM denotes the use of protein language model information. AUC is the area under the receiver operating characteristic (ROC) curve, and APS represents the average precision score along the precision‐recall curve. Cov refers to the proportion of the reference proteins the method successfully covers. Methods are sorted based on the AUC.

**FIGURE 3:**
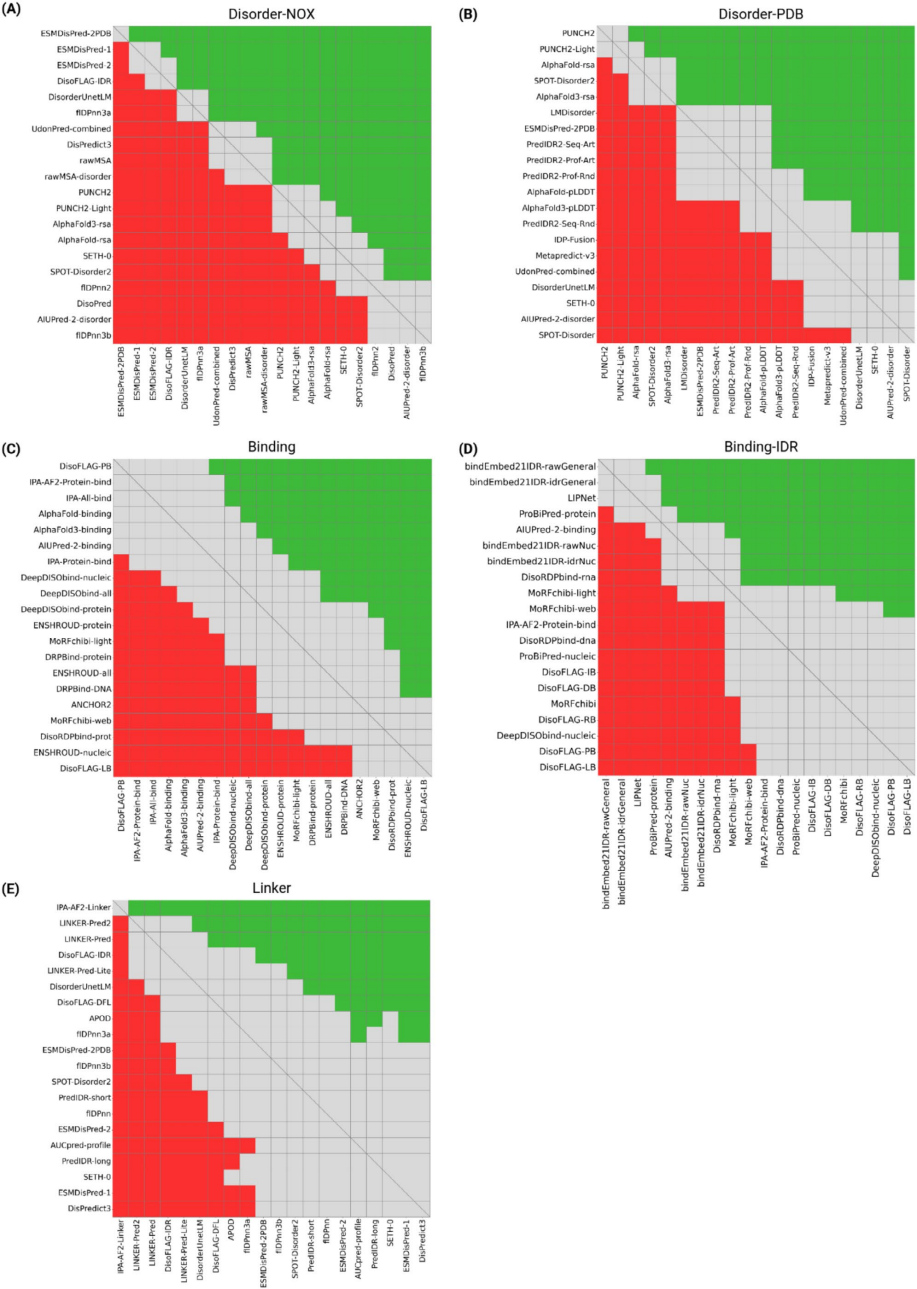
The heatmap of DeLong test results for the top 20 methods in (A) Disorder‐NOX, (B) Disorder‐PDB, (C) Binding, (D) Binding‐IDR, and (E) Linker references. The methods are ranked based on their AUC values.

The DeLong test results for Disorder‐NOX and Disorder‐PDB are visualized as a heatmap in Figure 3A,B, respectively. The green cells indicate a significant improvement in favor of the first predictor, the gray cells denote a non‐significant difference, and the red cells highlight a significant advantage for the second predictor. For example, in Figure 3B, PUNCH2 is not significantly better than PUNCH2‐light, but it outperforms all other predictors. The methods are sorted by AUC, complying with the rankings in Tables 1 and 2.

The varying performance of a method on Disorder‐NOX and Disorder‐PDB references likely reflects inherent differences in the datasets. The Disorder‐PDB dataset is a superset of Disorder‐NOX, including all proteins from Disorder‐NOX and additional proteins not present in the latter. For proteins common to both datasets, the Disorder‐PDB contains higher disorder (57.3% vs. 23.9%) and lower negative (order) content than Disorder‐NOX (75.3% vs. 79.2%). This occurs because sequence lengths decrease when negative instances are restricted to observed residues in PDB. However, the distribution of disordered residues remains nearly identical between the two datasets, indicating that DisProt annotations are supported by additional evidence codes (ECO) other than just “X‐ray missing residues”. Meanwhile, the extra proteins included in Disorder‐PDB primarily consist of short disorder fragments that skew the distribution of the count of disorder residues with 25.5% and 84.8% median content for positive and negative annotations, respectively (Figure 1E).

On the Disorder‐PDB reference set, AlphaFold‐rsa and AlphaFold3‐rsa rank in the top 10 (Table 2), but AlphaFold‐rsa does not show a significant advantage over AlphaFold3‐rsa when compared by AUC (Figure 3B). In contrast, AlphaFold‐pLDDT ranks 11th, while AlphaFold3‐pLDDT ranks 13th, making the pLDDT of AlphaFold2 structures more reliable when it is treated as the absence of structure for disorder prediction. Within AlphaFold3, the RSA‐based predictor outperforms its pLDDT‐based counterpart (Figure 3B), indicating that RSA may better capture disorder‐relevant structural features than pLDDT scores. Visual inspection of reference set structures reveals that AlphaFold3 often assigns higher pLDDT scores to regions where AlphaFold2 was less confident, which may explain the reduced performance of AlphaFold3‐pLDDT in disorder prediction.

### 3.2 Binding Sites and Linker Regions Prediction

The top 10 results of the Binding and Binding‐IDR challenges are presented in Tables 3 and 4, sorted by AUC. Due to label imbalance in the Binding reference (Figure 1D–F), predictors achieve higher AUC but lower APS compared to the Binding‐IDR reference (Tables 3 and 4), which is more balanced by definition (Figure 1D–F). This is because the large number of negative residues inflates the true negative rate in the Binding challenge. Additionally, because DisProt annotations are literature‐derived and potentially incomplete, some true binding residues may remain unannotated, leading to an apparent increase in false positives and a corresponding drop in APS. Top predictors in the Binding‐IDR challenge maintain a more stable precision compared to the Binding challenge (see website for PR curves) when recall increases, owing to a more balanced dataset. Despite this, the task remains challenging, with considerable room for improvement in both AUC and APS.

**TABLE 3:** Top 10 ranking of binding challenge.

Binding					
Predictor	New	pLM	AUC	APS	Cov
DisoFLAG‐PB [30]	×	×	0.776	0.245	0.980
IPA‐AF2‐Protein‐bind [38]	×		0.775	0.249	1.000
IPA‐All‐bind [38]	×		0.773	0.220	1.000
AlphaFold‐binding [24]	×		0.772	0.249	1.000
AlphaFold3‐binding			0.766	0.231	1.000
AIUPred‐2‐binding [39]	×		0.764	0.224	1.000
IPA‐Protein‐bind [38]	×		0.761	0.201	1.000
DeepDISObind‐nucleic [40]			0.756	0.237	1.000
DeepDISObind‐all [40]			0.754	0.188	1.000
DeepDISObind‐protein [40]			0.750	0.186	1.000

*Note*: New indicates whether it was submitted to CAID3. pLM denotes the use of protein language model information. AUC is the area under the receiver operating characteristic (ROC) curve, and APS represents the average precision score along the precision‐recall curve. Cov refers to the proportion of the reference proteins the method successfully covers. Methods are sorted based on the AUC.

**TABLE 4:** Top 10 ranking of binding‐IDR challenge.

Binding‐IDR					
Predictor	New	pLM	AUC	APS	Cov
bindEmbed21IDR‐rawGeneral [41]		×	0.641	0.514	1.000
bindEmbed21IDR–idrGeneral [41]		×	0.635	0.497	1.000
LIPNet	×	×	0.619	0.505	1.000
ProBiPred‐protein		×	0.613	0.472	1.000
AIUPred‐2‐binding [39]	×		0.590	0.463	1.000
bindEmbed21IDR‐rawNuc [41]		×	0.582	0.467	1.000
bindEmbed21IDR‐idrNuc [41]		×	0.579	0.459	1.000
DisoRDPbind‐rna [42]			0.575	0.450	1.000
MoRFchibi‐light [43]			0.564	0.421	0.980
MoRFchibi‐web [43]			0.548	0.419	0.980

*Note*: New indicates whether it was submitted to CAID3. pLM denotes the use of protein language model information. AUC is the area under the receiver operating characteristic (ROC) curve, and APS represents the average precision score along the precision‐recall curve. Cov refers to the proportion of the reference proteins the method successfully covers. Methods are sorted based on the AUC.

The top 10 rankings for the Linker challenge, sorted based on AUC, are available in Table 5. Since a limited number of methods are designed to predict linker regions, all the disorder predictors are evaluated for the Linker challenge. Although the rankings change when sorted by APS, the top method remains the same. As seen in the previous part, most of the top methods are the new methods participating in CAID3 and use protein Language Models (pLMs) to predict linker regions.

**TABLE 5:** Top 10 ranking of linker challenge.

Linker					
Predictor	New	pLM	AUC	APS	Cov
IPA‐AF2‐Linker [38]	×		0.897	0.474	0.903
LINKER‐Pred2	×	×	0.875	0.365	1.000
LINKER‐Pred	×	×	0.870	0.377	1.000
DisoFLAG‐IDR [30]	×	×	0.866	0.312	0.967
LINKER‐Pred‐Lite	×	×	0.854	0.393	1.000
DisorderUnetLM [31]	×	×	0.851	0.257	1.000
DisoFLAG‐DFL [30]	×	×	0.846	0.392	0.967
APOD [44]			0.843	0.321	1.000
flDPnn3a	×	×	0.841	0.228	1.000
ESMDisPred‐2PDB	×	×	0.836	0.345	0.806

*Note*: New indicates whether it was submitted to CAID3. pLM denotes the use of protein language model information. AUC is the area under the receiver operating characteristic (ROC) curve, and APS represents the average precision score along the precision‐recall curve. Cov refers to the proportion of the reference proteins the method successfully covers. Methods are sorted based on the AUC.

### 3.3 Comparison of CAID3 and CAID2

**FIGURE 4:**
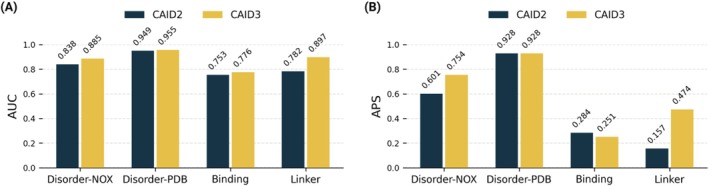
The AUC (A) and APS (B) comparison of the 1st place methods in CAID2 and CAID3.

The highest performance of the Disorder and Linker categories has improved in CAID3 compared to CAID2. The Linker challenge shows the most improvement, more than 11% and 31% in AUC and APS, followed by the Disorder‐NOX challenge, displaying an increase of more than 9% and 15% in AUC and APS, respectively (Figure 4).

Compared to CAID2, the fraction of negative residues in Disorder‐NOX has been reduced due to a change in the curation process in the DisProt database, mainly because the curators focus on collecting all relevant literature for a single protein before moving to the next target, rather than randomly searching new literature. With this approach, the number of targets has decreased, but the potential “incompleteness of the annotations” for a protein has improved drastically. Furthermore, for annotating the linker regions, curators take advantage of predictions of globular domains provided by InterPro [45], which are available in the DisProt curation interface. When a disorder region falls within two globular domains, the curators check experimental evidence of the globular regions and add an “IDPO:00502 flexible linker/spacer” annotation.

While the highest overall AUC in the Binding category has slightly improved from CAID2 to CAID3, the APS has decreased. This decline may be attributed to the stricter annotation filtering in CAID3, which excludes proteins with binding regions of undefined boundaries, which were previously associated with the entire underlying disordered region. As a result, identifying binding regions becomes more challenging, with reduced similarity to disorder reference sets.

### 3.4 Predicting Intrinsic Disorder by Protein Language Models

Protein Language Models (pLMs) learn the foundational knowledge of proteins from large‐scale pretraining on billions of sequences [14, 15, 16, 46]. These models encode the properties of residues and their context in high‐dimensional real‐valued representations called embedding. They could be directly used in protein‐related tasks [20, 47] or downstream problems in a transfer‐learning approach [41, 48, 49].

The use of pLMs has significantly increased in CAID3 compared to CAID2. In the latter, only five methods incorporated embeddings from pLMs as input for disorder prediction. In CAID3, 14 out of 24 methods incorporated different pLMs (Table 6), many of which topped the rankings (Tables 1, 2, 3, 4, 5, see the website for full results). This shift highlights the growing adaptation and versatility of pLMs in the field, driven by advancements in their design and accessibility.

**TABLE 6:** Usage of protein language models in CAID3 and CAID2.

	Predictor	ProtT5	ProstT5	ESM2	ESM‐1b	ESM‐MSA‐1b
CAID3	DisoFLAG [30]	×				
	DisorderUnetLM [31]	×				
	EBIND			×		
	ESMDisPred			×	×	
	LINKER‐Pred	×				×
	LINKER‐Pred2	×				×
	LINKER‐Pred_lite	×				
	LIPNet	×				
	LMDisorder [36]	×				
	PUNCH2 [34]	×				×
	PUNCH2_light [34]	×				
	UdonPred		×			
	fIDPnn3a			×		
	flDPnn3b			×		
CAID2	bindEmbed21DL [41]	×				
	DisPredict3.0 [32]				×	
	ProBiPred	×				
	SETH_0 [12]	×				
	SETH_1 [12]	×				

*Note*: ProtT5 refers to ProtT5_XL_UniRef50, ESM‐2 refers to esm2_t33_650M_UR50D, ESM‐1b refers to esm1b_t33_650M_UR50S models. Methods are sorted alphabetically.

In CAID3, the most commonly used model was ProtT5 [15] (9 methods), followed by ESM2 [46] (4 methods). Along with ESM‐1b [50], these models are single‐sequence‐based, that is, they require only protein sequence information without additional evolutionary data. This makes them particularly useful in cases where multiple sequence alignments (MSAs) are inadequate, for example in IDPs [51] or “Dark” proteome [52]. On the other hand, ESM‐MSA‐1b [53] is a multiple‐sequence‐based model that operates on MSA and has been used by two methods. ProstT5 [54] incorporates structural information by continuing ProtT5's training to translate between structure and sequence tokens.

The use of pLMs is most prominent in the Disorder‐NOX, Binding‐IDR, and Linker benchmarks, where the majority of top‐ranking methods rely on pLMs. In the Disorder‐NOX (Table 1) and Linker (Table 5) categories, pLM‐based methods outperform most of the previous CAID2 methods. Notably, in the Binding‐IDR benchmark, which focuses on identifying binding residues from IDRs, pLM‐based methods lead the rankings (Table 4). This suggests that pLMs effectively capture features associated with binding and intrinsic disorder.

## 4 Conclusions

The Critical Assessment of Protein Intrinsic Disorder (CAID) provides a fair and standardized platform for benchmarking IDR predictors. Beyond evaluating accuracy, CAID assesses practical aspects such as speed and runtime efficiency, key factors for large‐scale proteome predictions. By offering these insights, CAID helps researchers select the most suitable tool based on their specific needs and objectives.

To ensure high‐quality disorder annotations, CAID relies on the gold‐standard data from DisProt, a resource for manually curated annotations from literature. Over time, DisProt has improved its annotation process, which is reflected in the increasing proportion of disordered annotations in CAID3 compared to previous rounds.

The prediction of IDRs in CAID3 was evaluated using two approaches. Disorder‐NOX assumes that DisProt annotations (excluding X‐ray missing residues) are complete, considering any region not explicitly labeled as an IDR as ordered. Disorder‐PDB accounts for potential missing annotations by excluding negative residues that are not observed in PDB structures.

Additionally, the Binding and Linker sub‐challenges evaluate the methods for identifying the binding sites and linker regions as annotated in DisProt. In CAID3, we introduced Binding‐IDR, which uses the same proteins and positives as the Binding reference, but limits negatives to disordered non‐binding residues. Our results indicate that binding prediction remains challenging, partly due to data imbalance and also probably because current predictors have not yet reached their full performance potential.

In CAID3, significant improvements were observed in Linker and Disorder‐NOX, while the performance on Disorder‐PDB remained consistently high. In the Linker challenge, the best CAID3 method scored over 15% higher APS than the top CAID2 method. Overall, the predictors have progressed in disorder prediction. This is evident from many new methods achieving high scores across different performance metrics.

Finally, in CAID3, many methods incorporated embeddings from various protein Language Models (pLMs), most of them ranking in the top. This highlights the power of pLMs in capturing fundamental sequence properties, particularly those related to intrinsic disorder. By implicitly learning evolutionary information from sequence data, pLMs eliminate the need for multiple sequence alignments, enhance prediction accuracy, and computational efficiency in predicting intrinsic disorder.

Complete results for CAID3 are available on the challenge website: https://caid.idpcentral.org/challenge/results. Additionally, the CAID prediction portal (https://caid.idpcentral.org/portal) allows users to run most CAID methods on their own sequences, compare predictions and evaluation metrics, and select the methods that best match their accuracy needs and computational resources.

## Author Contributions

**Mahta Mehdiabadi:** conceptualization, investigation, writing – original draft, software, methodology, validation. **Alessio Del Conte:** investigation, software, methodology, validation. **Maria Victoria Nugnes:** data curation. **Maria Cristina Aspromonte:** data curation. **Silvio C. E. Tosatto:** conceptualization, funding acquisition, methodology, writing – review and editing, supervision. **Damiano Piovesan:** conceptualization, investigation, writing – original draft, writing – review and editing, methodology, funding acquisition, supervision, software.

## Data Availability

The code to produce references and dataset statistics is available in the caid‐reference GitHub repository at URL: https://github.com/BioComputingUP/caid‐reference. Results of the CAID assessment can be fully reproduced by downloading the code and following the instructions in the CAID GitHub repository at https://github.com/BioComputingUP/CAID. Some CAID methods are available as web services in the CAID Prediction Portal at URL: https://caid.idpcentral.org/.” [Correction added after first online publication on 25 September 2025. The Data Availability Statement was updated].
